# Reflections for the Future of AMR Surveillance and AMR Action

**DOI:** 10.1093/cid/ciag498

**Published:** 2026-10-19

**Authors:** Clare I R Chandler, Syed Abbas, Afreenish Amir, Sylvia Bertagnolio, Lillian Bibangamba, Colin Brown, Hannah Brown, Roderick M Card, Adrienne Chattoe-Brown, Rachel Dalton, Archie Drake, Stanley Fenwick, Clare Gilbert, Claire Gordon, Ankur Gupta-Wright, Heidi Hopkins, Emmanuel Kabali, Freddy Kitutu, Toby Leslie, Rebecca Maudling, Maxencia Nabiryo, Amy Potter, Helena Rosado, Victoria Rutter, Nandini Shetty, Tim Shorten, Milena von und zur Mühlen, Catriona Waddington, Jaap A Wagenaar, Evelyn Wesangula, Dan Whitaker, Javier Yugueros-Marcos, Kate Macintyre

**Affiliations:** London School of Hygiene and Tropical Medicine, United Kingdom; Fleming Fund Technical Advisory Group; Institute of Development Studies, University of Sussex, United Kingdom; Itad Ltd, Independent Consultant, International House, Queens Road, Brighton, United Kingdom; Fleming Fund Technical Advisory Group; Department of Antimicrobial Resistance, World Health Organization; Fleming Fund management agent, Mott MacDonald, London, United Kingdom; UK Health Security Agency; Itad Ltd, Independent Consultant, International House, Queens Road, Brighton, United Kingdom; Animal and Plant Health Agency; Fleming Fund management agent, Mott MacDonald, London, United Kingdom; Food and Agricultural Organization; Itad Ltd, Independent Consultant, International House, Queens Road, Brighton, United Kingdom; Fleming Fund management agent, Mott MacDonald, London, United Kingdom; College of Environmental and Life Sciences, Murdoch University, Australia; Veterinary Medicines Directorate; Fleming Fund Technical Advisory Group; UK Health Security Agency; Itad Ltd, Independent Consultant, International House, Queens Road, Brighton, United Kingdom; Department of Infectious Disease, Imperial College London, United Kingdom; Department of Infectious Diseases and Tropical Medicine, University of Heidelberg, Germany; Department of Infectious Diseases, North Bristol NHS Trust, Bristol, United Kingdom; Fleming Fund management agent, Mott MacDonald, London, United Kingdom; Food and Agricultural Organization; Department of Pharmacy, Makerere University School of Health Sciences, Kampala, Uganda; Department of Women's and Children's Health, International Child Health and Migration, Uppsala University, SE-751 85 Uppsala, Sweden; Fleming Fund management agent, Mott MacDonald, London, United Kingdom; Itad Ltd, Independent Consultant, International House, Queens Road, Brighton, United Kingdom; Commonwealth Pharmacists Association; Foreign Commonwealth & Development Office, United Kingdom; Commonwealth Pharmacists Association; Commonwealth Pharmacists Association; Department of Health and Social Care, United Kingdom; Itad Ltd, Independent Consultant, International House, Queens Road, Brighton, United Kingdom; Department of Health and Social Care, United Kingdom; Fleming Fund Expert Advisory Group, United Kingdom; Fleming Fund Technical Advisory Group; Division of Infectious Diseases & Immunology, Faculty of Veterinary Medicine, Utrecht University, The Netherlands; Itad Ltd, Independent Consultant, International House, Queens Road, Brighton, United Kingdom; Family Health and Infectious Diseases, East Central and Southern Africa Health Community, Arusha, Arusha, Tanzania; Itad Ltd, Independent Consultant, International House, Queens Road, Brighton, United Kingdom; World Organization for Animal Health; Itad Ltd, Independent Consultant, International House, Queens Road, Brighton, United Kingdom; Oxford Health Evaluations, Buckingham, United Kingdom

**Keywords:** antimicrobial resistance, lessons learnt, future steps

## Abstract

**Background:**

This paper builds on the other papers in this series to present high-level lessons learnt and considerations for the future of antimicrobial resistance (AMR) surveillance and its contribution to action on AMR in low- and middle-income countries.

**Methods:**

Participatory synthesis involved engagement with individuals who had worked within and alongside the Fleming Fund from a range of organizations and geographies, to scope and solidify lessons learnt and future considerations relevant to a wide range of donors, partners, and policy makers or implementors. Contributors were invited to join a Writing Group, which included individuals from academia, clinical sciences, applied public, animal, environment, and food sciences health, as well as senior scientists and administrators from 14 organizations. The synthesis was informed by contributions to 4 virtual workshops and 3 drafts of the manuscript. Authors read and commented on the drafts, several wrote and referenced sections, and all gave substantive feedback with their reactions to the draft conclusions as consensus was built.

**Results:**

Over nearly 10 years, the Fleming Fund achieved significant improvements in AMR surveillance. Analysis of the barriers and enablers across settings and systems highlights key lessons for how surveillance is positioned, funded, used, and innovated upon. The success of future AMR surveillance and its sustainability relies on planning within resource constraints for a sharpened system that focuses the scope, frequency, and depth of data to inform action, technical, and architectural developments directions to streamline investment and financing that supports country-led sustainable approaches.

**Conclusions:**

Investment in AMR surveillance and broader AMR actions has yielded substantial progress. Expanded domestic public financing, large-scale private sector engagement, or enhanced multilateral support will be needed to ensure recent gains are sustained. Progress will require well-designed, targeted, and cost-effective investments to maintain current capacity while enabling further system strengthening.

This article is part of a journal series synthesizing the evidence and lessons from the Fleming Fund to contribute to wider learning on how complex global health programs translate technical capacity into institutional and policy change, and to strengthen the use of evaluative approaches within UK-funded antimicrobial resistance (AMR) programming and the wider literature.

After a decade of implementation and investments up to £498 million, the Fleming Fund has achieved many of its original objectives [1, 2]. These include strengthened AMR surveillance systems through the renovation and equipping microbiology laboratories, training of laboratory workforce, and the reinforcement of advocacy, stewardship, and governance in clinical services and across sectors thereby expanding AMR data availability and use. The program has increased awareness and knowledge management at multiple levels, from community initiatives, such as Food and Agricultural Organization's (FAO’s) Farmer Field Schools [3], to guiding the development of national action plans (NAPs) and multisectoral governance arrangements for AMR management in >20 low- and middle-income countries (LMICs), to inputs such as guidelines and local and global interactive online platforms for data sharing (eg, the World Health Organization’s [WHO's] Global Antimicrobial Resistance and Use Surveillance System [GLASS] [4], InFARM [5], and ANIMUSE global databases) [6].

Yet, in part because of the interconnectedness of the AMR challenge, the work is unfinished. AMR is acknowledged as a “wicked” problem [7] and the world continues to experience AMR as a major health, food security, and economic threat to societies everywhere [8, 9]. The global burden is substantial, with an estimated 1.14 million deaths attributable to bacterial AMR in 2021 [9], and a broader economic impact projected to reach trillions of dollars in cumulative losses over coming decades [10]. Finance ministries as well as health ministries have a stake in these outcomes.

The rising health system and productivity losses associated with AMR mean that decisions on whether and how to fund surveillance are no longer purely technical but central to priority-setting and fiscal policy. The cost of inaction (see Figure 1) includes threats to health systems that can dramatically increase the costs to society, from increased disability and reduced productivity, increased hospital stays, risks to surgery, and rises in morbidity and early mortality (Figure 2) [10]. The impact of AMR disproportionately affects those already facing health inequalities, with the burden higher in LMICs [12]. At the population level, there is increased impact on the young (younger than 5 years) and the elderly (older than 70 years), with evidence of additional gender disparities [13–15]. For livestock productivity, the losses are already huge, with AMR causing annual gross domestic product (GDP) losses of US$17 billion across livestock systems [16]. By 2050, if we do not change behavior, annual global livestock GDP losses will reach up to US$1.7 trillion [17].

**Figure 1.**
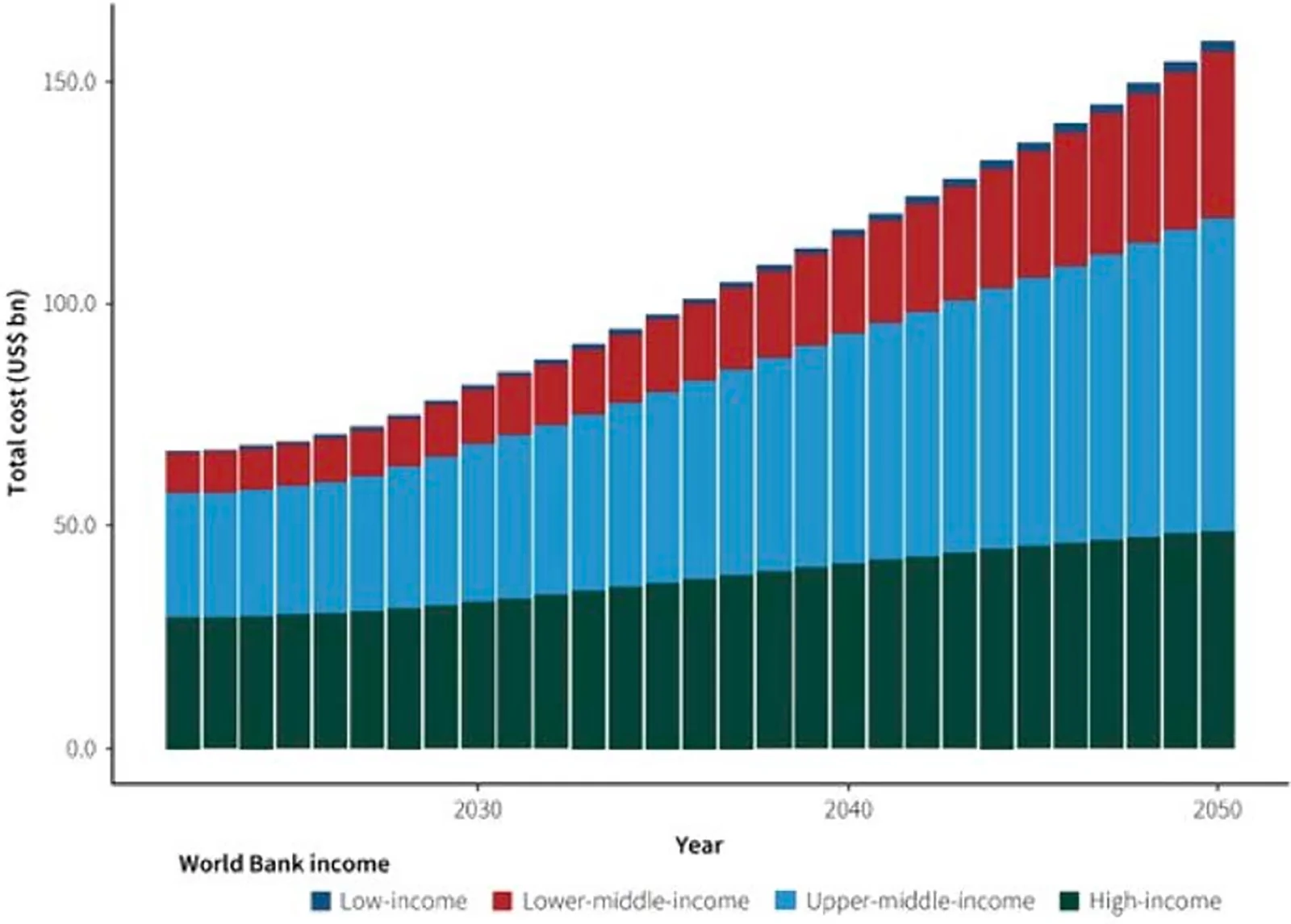
Global increase in excess inpatient health care costs associated with AMR, 2020–2050. Source: Forecasting the Fallout from AMR: Economic Impacts of Antimicrobial Resistance in Humans. Center for Global Development [11].

**Figure 2.**
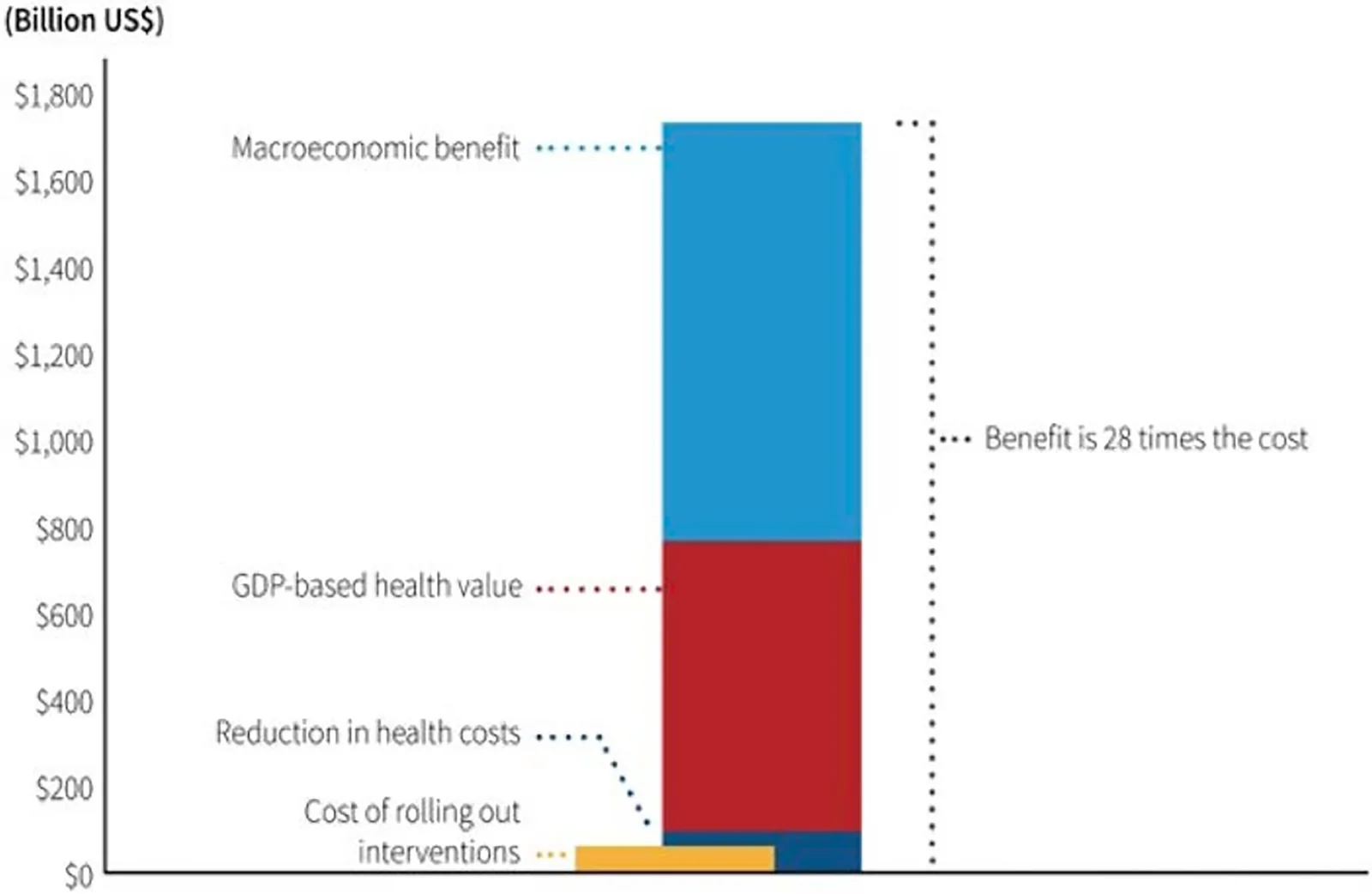
Estimated annual costs and benefits in 2050 from better treatment for bacterial infections and innovative new gram-negative drugs.

Projections indicate that well-designed surveillance system investments can yield strong economic returns, characterized by favorable benefit–cost ratios and high cost-effectiveness [18, 19]. This illustrates that AMR surveillance is not only a core component of an effective public and animal health response but a strategic economic investment, which if acted on can mitigate productivity losses, reduce long-term healthcare expenses, and strengthen resilience across health and food production systems.

A central challenge for AMR surveillance is no longer whether quality data can be generated (even if high-quality data are still not produced everywhere), but how surveillance systems can be designed to reliably inform action under conditions of constrained financing, competing priorities, and institutional complexity. Drawing on the Fleming Fund experience and the experience of using GLASS findings, we examine what enables AMR surveillance to be translated into action, and how surveillance functions can be better structured to be sustainable. Fleming Fund’s experience stretched across 25 countries as well as at regional and global levels. The focus countries included: Sierra Leone, Ghana, Senegal, Zambia, Tanzania, Kenya, Bhutan, Bangladesh, Indonesia, Laos, Pakistan, Nigeria, Uganda, Nepal, Timor-Leste, Vietnam, Myanmar, Zimbabwe, India, Sri Lanka, Malawi, Eswatini, and Papua New Guinea. (Other countries, beyond those listed, were supported through other grant mechanisms such as the Antimicrobial Resistance Multi-Partner Trust Fund or work in the Caribbean). We reflect on lessons about how a One Health approach can be applied to conducting AMR Surveillance across human, animal and environment sectors.

The constraints facing surveillance are substantially different now compared to 2015 when the Fleming Fund was conceptualized. Following a brief discussion of recent shifts in the financial and political landscape, and an overview of critical insights from the supplement's 4 papers (Box 1), we consider future surveillance based on the lessons learnt, with relevance for development partners and national policymakers.

This paper forms part of a practice-informed series synthesizing learning from Fleming Fund–supported AMR surveillance strengthening activities. The paper used a participatory synthesis approach [20]; drawing on program documentation, evaluation evidence, relevant literature, and structured engagement with a broad range of contributors with direct involvement in or alongside Fleming Fund activities, including implementing countries and the wider AMR community. The series does not constitute an independent evaluation or seek to establish causal attribution. Findings should therefore be interpreted in light of limitations including program-linked authorship, contributor perspectives, and purposive source selection.

The group developed the analysis iteratively through a series of 4 virtual workshops interspersed with reviews of draft content. The writing group (composed of 33 experts) represented a range of disciplines: public health, clinical infectious diseases, veterinary medicine, microbiology, food/nutrition sciences, pharmacy, epidemiology, international development, health policy, implementation and evaluation practice, behavioral science, and health and surveillance systems. All authors commented on the papers; some contributed by drafting and referencing specific sections. The group, which included individuals from across 4 continents, reflected representation from multilateral organizations, government agencies, universities, development consultancies, nongovernmental organizations, and professional associations, illustrating the breadth of expertise required to address AMR across a One Health approach.

## THE FLEMING FUND IN THE GLOBAL CONTEXT

In 2015, the WHO published the Global Action Plan (GAP) on AMR [17], which was endorsed by the World Health Assembly and later reinforced by a landmark Political Declaration adopted by the UN General Assembly in 2016. Surveillance and research were enshrined as 1 of 5 strategic objectives of the GAP and alongside awareness, infection prevention, optimized antimicrobial use, and sustainable investment. At the time, the most significant gaps in surveillance capacity were concentrated in LMICs [21]. The establishment of a dedicated AMR division within WHO oversaw the launch of GLASS. By the end of 2024, 127 countries and 3 territories/areas had joined GLASS and 104 countries reported data in 2023 [4]. The GAP catalyzed a cross-sectoral institutional response, with the formalization of a tripartite collaboration between WHO, FAO, and World Organization for Animal Health (WOAH) in 2018, followed by inclusion of United Nations Environment Programme (UNEP) to form a quadripartite in 2022. In the animal health sector, FAO and WOAH significantly increased their resourcing and internal capacity on AMR. With Fleming Fund investment, the headcount of individuals working on AMR or AMR-adjacent work increased and digital platforms were developed: WOAH developed ANIMUSE, which monitors antimicrobial use in animals, and FAO launched the InFARM program [5], a global information system for AMR in livestock, aquaculture, and food and antimicrobial use (AMU) in plant production. The 2024 UNGA High-Level Meeting on AMR saw member states make a second political declaration, with further commitments on AMR, including on strengthening surveillance, across animal, agricultural, environmental, and human health [22]. The UNGA declaration signposted the quadripartite guidance on One Health integrated surveillance of AMR and AMU (GISSA), launched in 2026 to support cross-sectoral surveillance [23]. These developments across the quadripartite, as well as within countries as discussed next, were in part emergent through Fleming Fund investments.

The significance of surveillance emerged against a backdrop of growing concern about the potential future scale of AMR, together with recognition of data uncertainties.

In the United Kingdom, in 2016 the O’Neill Report on AMR outlined the scale of the AMR problem—the loss of 10 million lives a year globally by 2050 if urgent action was not taken [24]. It proposed several actions, and the Fleming Fund was developed to directly address the recommendations, particularly the lack of AMR data in LMICs, the need for laboratory capacity strengthening, support for One Health governance, and the implementation of NAPs. The structure and approach taken by the Fleming Fund along with some of the outcomes are outlined in the earlier publications in this series.

The Fleming Fund's closure in 2026 comes amid historic reductions in global Overseas Development Aid from many donor nations. This cost-cutting from donors is projected to cause 9.4 million excess deaths if current downward trends continue [25]. In this context of tight budgets, surveillance is harder to argue to protect than more tangible services such as hospital beds or medicines, despite its central role in preventing much larger downstream costs. These uncertainties produced by abrupt reductions in resources could worsen AMR, through limited access to medication and diagnostics, dissolution of programs to improve water, sanitation and hygiene, vaccination, and reducing funding for One Health approaches that protect against AMR by incorporating food, agricultural, animal, and environmental systems as well as human health systems [26]. The 2023 Lusaka Agenda's recommendations involve greater health system strengthening including integrated primary healthcare services, and a gradual transition toward sustainable, domestically financed health services and public health functions. However, this transition still needs support. In many countries, AMR surveillance remains “critically underfunded, undermanned and under-resourced” [27], which undermines the quality, relevance, completeness, and actionability of the data generated [4]. This context is not entirely new and does not necessitate inertia; what is required is clear planning for surveillance design choices, informed by experience across settings and systems.

Recognizing the intersection between AMR and the disease burdens addressed by the Global Fund, WHO has issued dedicated guidance encouraging countries to include AMR surveillance and laboratory strengthening activities in their Global Fund grant proposals, noting that such investments support more effective human immunodeficiency virus, tuberculosis, and malaria programs while simultaneously building broader infectious disease preparedness [12]. AMR surveillance and laboratory strengthening activities are therefore eligible for inclusion in Global Fund Grant Cycle 8 proposals, offering countries an important pathway to strengthen national surveillance capacity within existing grant mechanisms [28]. The Pandemic Fund further recognizes AMR as the “silent pandemic” and includes it in its One Health funding for pandemic prevention, preparedness, and response [29]. The animal health sector is increasingly promoting the inclusion of AMR surveillance in food safety programs, with emphasis on a farm-to-fork continuum [30].

AMR surveillance generates nonexcludable information that improves clinical care, guides stewardship, and reduces cross-border risks, giving it the characteristics of a global public good and a foundational public health function. Yet in many LMICs, surveillance investments compete with other pressing health priorities, and high out-of-pocket spending on AMR-related care exposes poorer households to catastrophic costs and income loss. Underinvesting in surveillance is therefore regressive and risks widening existing inequities, reinforcing the case for financing models that couple sustained domestic commitment with predictable international cofinancing and explicitly consider who benefits from better AMR information.

## LESSONS FROM THE FLEMING FUND

During its implementation, the Fleming Fund supported a wide range of institutions to operationalize different aspects of AMR surveillance. Grants evolved from a core of laboratory infrastructure, training, and implementation to encompass a range of policy, economics, quality assurance, and mentorship activity at multiple levels of a global AMR ecosystem. The Fleming Fund brought together different worlds—not only across One Health domains and across nations, but notably the worlds of microbiology and of development. In its evolution, it began to develop bridges between these worlds. Many questions and lessons emerged at this intersection, around whether and how the systems for surveillance can be embedded and sustained, and how they can produce representative and actionable data at reasonably low cost. This section unpacks these issues in the context of program closure and lessons learnt.

Box 1 summarizes each of the supplement papers' key messages that can translate into more forward-looking actions. These messages include the background and springboard for the considerations laid out below.

Box 1. **Presenting summary messages from the four other papers in the supplement legacy series**

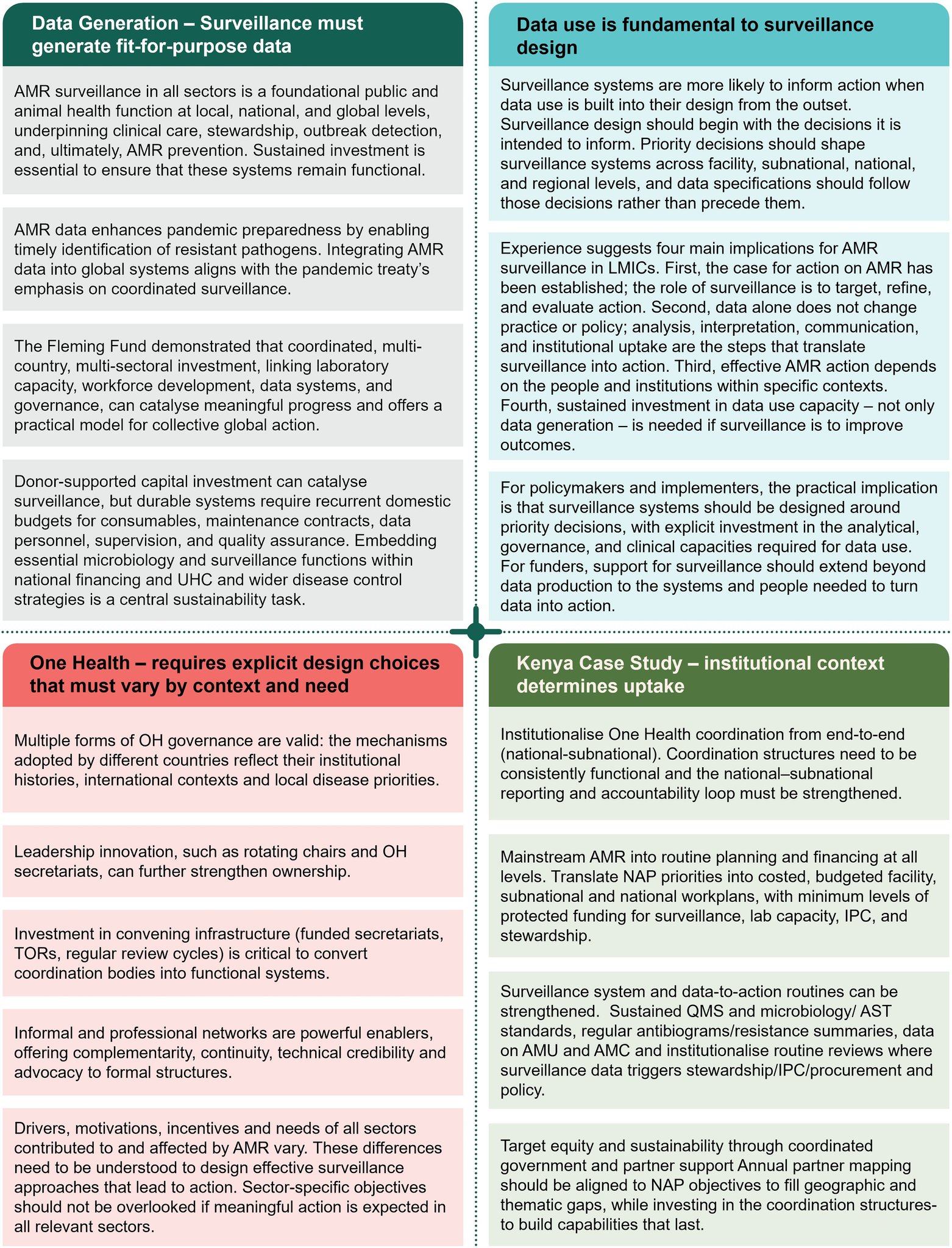

Barriers and enablers observed across Fleming Fund countries: An analysis of the enablers and barriers to sustaining the AMR Surveillance in LMICs grouped factors under: (1) government ownership and budget allocation, (2) technical capacity built and retained, and (3) AMR data use integrated within national systems [31]. These factors were drawn from country reporting during the project and during various evaluations and synthesized into a report published by the Fleming Fund Knowledge Hub [31]. These factors were discussed extensively by the Writing Group members, but they were not formerly ranked for priority or impact as many of them are highly dependent on country contexts. Table 1 shows the factors that emerged from this analysis.

**Table 1.** Barriers and Enablers Observed Across Fleming Fund Countries

Theme	Enabling Factors	Barriers/Challenges
Government ownership and budget allocation	High-level political champions Economic evidence Multisectoral approach Decentralized AMR governance and budgeting Public financial management (PFM) Costed National Action Plan AMR activities incorporated into government systems Integration of microbiology diagnostic tests into national health insurance schemes Models/plans for equipment maintenance National and international reporting obligations	Competing priorities Cost of consumables, reagents service agreements and maintenance Quality of diagnostic test results Proprietary branded consumables Cost of active surveillance in AH sector Vertical funding of AMR outside government systems Changes in funding landscape Lack of planning for sustainability
Technical capacity built and retained	Integration of AMR into preservice education National leadership and mentorship programs/communities of practice Trust in data quality Future scaling—quadripartite (WHO, FAO, WOAH, and UNEP)	Brain drain Human resource turnover Lack of private sector involvement Lack of dedicated time commitment
AMR data use integrated within national systems	Multisectoral stakeholder collaboration Interoperable digital data systems Primary health care reform Antibiotic stewardship and inclusion of private sector in surveillance Domestic demand for data Availability of protocols to guide AMR and AMU data collection, such as WHO and global PPS methodologies	Shortage of time for implementation Difficulty regulating AMC and AMU Reluctance or difficulty sharing data Vertical approaches not affordable Lack of methodological harmonization of AMR and AMU data collection Funding for AMR surveillance

Abbreviations: AH, animal health; AMC, antimicrobial consumption; AMR, antimicrobial resistance; AMU, antimicrobial use; FAO, Food and Agricultural Organization; PPS, Point Prevalence Survey; WHO, World Health Organization; WOAH, World Organization for Animal Health; UNEP, United Nations Environment Program.

**Country-led trajectories for sustainability:** The future trajectory of AMR surveillance and action will hinge on aligning global and country ambitions with the political, technical, and financial capabilities required for sustained system performance. Evidence from the Fleming Fund illustrates considerable heterogeneity in national progress; whereas some countries have translated investments into strengthened governance and laboratory capacity, others face foundational constraints, underscoring the need for approaches that are context-specific, financially feasible, and genuinely country-led [32].

**Donor-Led Approaches Shape Long-Term System Sustainability:** Reliance on external sources during the foundational years of system development without genuine efforts to combine domestic commitment, resource mobilization, and investment, can constrain the adaptability and long-term sustainability of local systems. Donor-led approaches, particularly those involving extensive engagement of external technical teams in building national systems, may inadvertently weaken institutional ownership and decision-making capacity within countries, hindering transition toward fully autonomous, locally governed, and embedded systems [33].

**Vertical systems are fragile in most contexts:** Concurrent shifts in the global health architecture—including the drive for integration articulated in the Lusaka Agenda, alongside the decline in donor funds for health and increasing discussions around sovereignty and domestic financing [34, 35] highlight the limits of vertical, siloed investments, including for AMR. Disease- or test-specific laboratory gains have often outpaced development of the demand, financing, and institutional structures that are needed to sustain broader systems.

Lessons demonstrate that laboratory-focused models, although essential to generating AMR data, are inherently resource intensive and vulnerable if not embedded within wider surveillance and health system functions. Where ongoing costs for equipment, reagents, and maintenance exceed the resources that can be sustained domestically, such models are at greater risk of underuse once external funding reduces, underscoring the importance of early design choices, transition planning, and explicit dialogue with governments about affordability. The question of whether gains will be sustained without ongoing AMR-specific investment, particularly into equipment maintenance, purchase of reagents, and laboratory consumables underscores the need to integrate AMR into national surveillance systems under a holistic national health sector plan [36] rather than addressed on its own [37, 38]. These considerations are equal, if not more pertinent, when considering AMR in animal health, which has tended to be underfunded, with even weaker underlying systems.

**Multisectoral approaches:** The importance of comprehensive and multisectoral approaches that address the drivers of AMR within and beyond the health system was underscored by many lessons from the Fleming Fund (WASH, IPC, vaccinations, timely access to quality health care including antibiotics, etc) [39]. Cross-sectoral governance for AMR surveillance both at national level and at facility level, and beyond human health has produced myriad lessons (see Paper 4). But meaningful surveillance depends not only on laboratory outputs but also on the capacity of human, animal, and environmental sectors to collaborate, prioritize and track how investments are delivering outcomes [23].

**Integrating nonhuman health sectors into One Health surveillance programs:** Although One Health has been a guiding principle of the Fleming Fund, in practice it has been difficult to integrate all sectors into long-term, sustainable surveillance programs. There are a few reasons for this—first, the lack of awareness that AMR is an issue for the animal and environment sectors at local levels, as until relatively recently it has been largely framed as a human health problem; and second, the lack of resources for the nonhuman sectors to implement public health surveillance, with many competing priorities from animal diseases to water quality. There exist structural differences in current systems and differing pressures, needs, and incentives. Progress has been made; however, there needs to be more support and time for these sectors to build AMR surveillance into more cost-effective systems including those already in place (eg, food safety, meat hygiene, disease surveillance programs, water supply testing), to support analysis, and to promote cross-sectoral control and mitigation programs [39].

**Generating Demand:** Experience from the first phase of the Fleming Fund reinforced the point that generating high-quality data is insufficient if the aim is to create a functional and sustainable passive surveillance system. The need to understand who requires the data and for what purpose leads to more appropriate, cost-effective, and equitable data at multiple levels of operation. If analysis and the data produced are aligned with the needs and priorities of the countries, then the decision makers can make better use of those data for improving outcomes [38]. The need to understand the countries' or institutions' capacity, authority, and processes to interpret and act appropriately is essential, as is an understanding of the barriers. For instance, asking questions about whether relevant, competent committees and frameworks of operation exist? Whether guideline-developing bodies and regulatory mechanisms are functional? Effective systems need feedback loops where data inform action, and action then refines future priorities.

**Linking AMR and AMU to develop policies for action:** much of the focus of the Fleming Fund has been on AMR surveillance, with support for AMU/antimicrobial consumption surveillance receiving less concerted focus. This remains an important consideration, not least to be able to differentiate and restrict use of antimicrobials of critical importance in humans—see Box 1 [32] as well as to monitor the effects of action on both AMU and AMR. Where action is needed in nonhuman health sectors emphasis needs to be given to designing systems that meet sectoral needs. Across sectors, countries need to understand which antibiotics are used and why, to inform stewardship, standard treatment guidelines and essential medicine lists [10, 32, 40].

## CONSIDERATIONS FOR THE FUTURE

Choices on structuring and sustaining surveillance gains will occur within substantial resource constraints, domestic and international. Here, technical, resource and practical considerations are combined to support prioritization within future constraints.

### Surveillance for Action

Surveillance data can inform a range of actions at different scales (see Figure 3) [39]. To inform these actions, the nature of data required and the pathways for data vary. Treatment and prevention need different data, and denominators, to outbreak or AMR emergence detection. Identifying at-risk populations, hotspots, and evaluating programs need additional data to those collected for immediate clinical decisions. Advocacy may need less granular data. A risk for surveillance is for it to be expected to produce a dataset with all relevant information for all questions and potential actions. The reality of implementing surveillance in constrained human or animal health systems with other pressing priorities obliges a targeted approach. The question of “what are the minimal essential data required for X action?” can help focus future efforts. Some actions will be equally well informed by one-off surveys (for example the Point Prevalence Studies in hospitals) [41] or in-depth studies and some may not be changed by surveillance data. Starting from actions and working backwards to consider the role and nature of surveillance required could be beneficial for refining the scope, frequency, depth, and data pathways.

**Figure 3.**
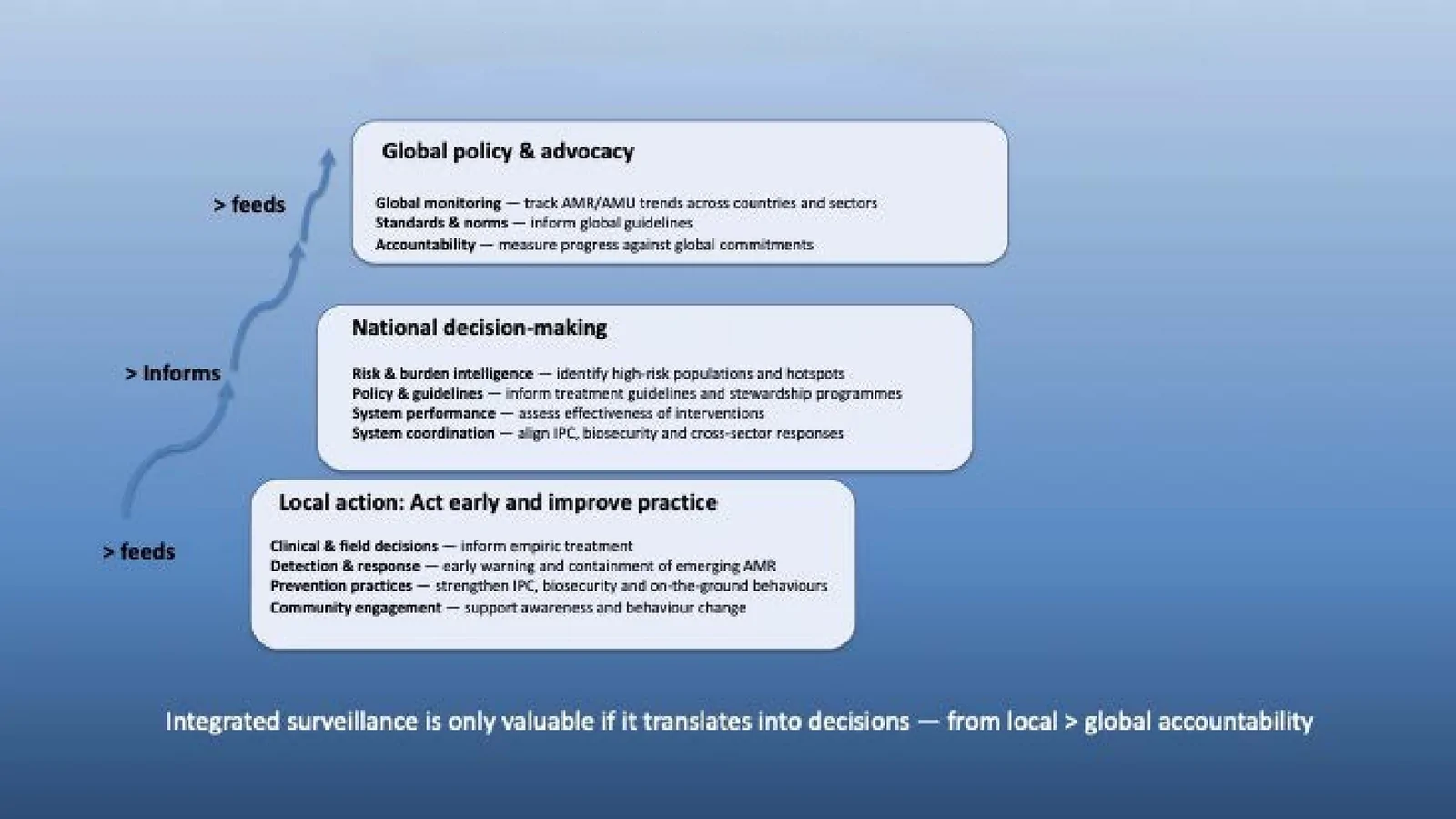
How integrated AMR & AMU surveillance drives decisions from local action, national decision-making to global policy.

Guidelines, and supporting behavior change, connected by arrows guiding information flow.

**Scope.** To inform anti-biograms, denominator data for medicines and supplies is required. This information is crucial for understanding both drug susceptibility and resistance. It helps to determine which drugs remain effective locally and allows for periodic data interpretation, unless unusual patterns emerge [1, 38].

**Frequency.** The full range of activity under surveillance and data generation may not need to be continuous. For example, event-based surveillance can be more responsive, with investment in ensuring good communication when clinical events are flagged and readiness to stand-up processes for investigation.

**Depth.** Understanding at-risk populations and hotspots requires gathering additional information about the patients/animals/farm/health facility, and although surveillance should routinely include some of this information, it could be that research studies need to take on these kinds of analyses. This should link to academic institutions.

**Data pathways.** Specific actions can simplify data flow, such as linking resistance profiling to medical supply requirements.

Such an approach requires a starting point of working with those in a position to take actions to impact AMR: forming an understanding of demand, and potentially generating demand, for data to inform action. As demand is defined for different groups, crops, and animals, it will be essential to establish clear, measurable targets as without them, implementation lacks direction and accountability. Building on the lessons provided here, this section sets out how AMR surveillance can be more deliberately designed and prioritized—across actions, architectures, and financing—to ensure that limited resources generate data that drive better decisions.

### Future Technical and Architectural Directions

A key purpose for AMR surveillance is to support estimates of the burden of disease for populations. To date, the focus has been on collection and analysis of isolate level data (ie, generating proportions of resistance in clinical isolates). In health, calls for linking these data to patient attributes have been powerful; knowing who these individuals are can improve understandings of risks including in different demographic groups such as by geography, gender, and age [14, 42, 43], which can help to inform AMR burden and target interventions correctly^14^; estimates rely on models for mortality and morbidity [44]. For countries to gain a better appreciation of who is shouldering the burden of AMR and in what ways, it will be important to include—perhaps through periodic add-on studies that enhance surveillance—follow-up data on survival, recovery rates, the duration and nature of clinical, economic and social impacts for patients with drug-resistant infections, their families, the health system at large, and the workforce.

In animal health, there is a gap in understanding circulating pathogens and resistance profiles. Indeed, to support the responsible and prudent use of antimicrobials in the animal health sector, data related to the health of animals are needed. This requires laboratory capability to diagnose endemic animal diseases driving antimicrobial use (which may or may not be bacterial). Surveillance focused purely on public health data results in limited understanding of circulating pathogens, resistance profiles and of the drivers of antimicrobial usage in animals.

Technological advances may further strengthen antimicrobial diagnostics and stewardship [45], particularly in relation to digital maturity and data integration. Although LIMS and data analysis platforms such as WHONET have improved standardization, interoperability with clinical records, antimicrobial consumption datasets, and emerging molecular or genomic platforms remain limited in scale and accessibility across most settings. This is often cost-driven by appetite for newer technologies.

Artificial intelligence and advanced analytics offer potential for real-time AMR prediction and clinical decision support [10]; yet, limits in data quality and completeness continue to constrain their implementation [46]. Technological feasibility is nonetheless advancing rapidly. For example, whole-genome sequencing and the establishment of regional genomic hubs have expanded in several LMICs, supported in part by investments associated with the coronavirus 2019 pandemic response and initiatives such as the Fleming Fund [47]. Despite this progress, the integration of genomic data into routine surveillance systems and national reporting frameworks remains at an early stage [48].

Good quality and efficient data management is essential at multiple levels, from major global conversations regarding information sovereignty, to community and national data interoperability. Between these lie complex issues of data ownership, privacy protocols, domestic legal frameworks, cross-border data-sharing agreements, and the distinct governance requirements of genomic surveillance architectures.

Further innovations—including microfluidics, molecular diagnostics, decentralized point-of-care testing, and cloud-based analytics—hold promise. But, again, without concurrent strengthening of demand generation, data collection, storage, and operational systems, as well as quality management and sustainable financing, the deployment of such technologies risks exacerbating existing inequities [49].

However necessary, technical and technological advances are insufficient to embed AMR surveillance effectively. Future AMR surveillance efforts will need to move beyond predominantly vertical approaches, instead embedding surveillance within integrated, multidisease platforms that align with routine health system workflows and universal health coverage strategies, thereby enhancing efficiency and long-term sustainability [50].

The convergence of microbial food safety and AMR represents a growing threat to food security and safety. But there are complex dynamics of foodborne AMR, including key points of emergence, transmission, and amplification across human, animal, and environmental interfaces. The widespread misuse of antimicrobials in agriculture, aquaculture, and clinical settings have driven the dissemination of multidrug-resistant pathogens, including *Salmonella* spp., *Campylobacter*, and *Escherichia coli*, throughout the food chain.

Addressing this will require a One Health approach that integrates antimicrobial stewardship, surveillance, regulatory oversight, innovation, public engagement, and strong cross-sectoral coordination mechanisms [51]. Advances in molecular diagnostics, whole-genome sequencing, and resistome analysis have improved the detection of resistance. And alternative strategies such as bacteriophage therapy, bacteriocins, and probiotics offer the potential to reduce reliance on conventional antimicrobials in food production systems. However, these advances must be matched by equitable access, particularly in LMICs where laboratory capacity remains limited. Optimizing AMU in livestock sectors will require the ability to implement effective husbandry practices and strong biosecurity measures including cleaning and disinfection; timely access to diagnostics, medicines and veterinary services; and implementation of vaccination programs.

Several authorities are now focused on the impact of climate change and AMR, with increasing evidence of association. As yet, the United Kingdom's is the only NAP to explicitly mention this as a commitment [52]. Tackling this as interconnected syndemics will require newly linked datasets and expanded analytical processes.

### Financing and Sustainability

In many ways, AMR is a classic health system strengthening problem, requiring investments in system design, data science, modeling and economic evaluation, surveillance, laboratory, workforce development, bioinformatics, behavioral science, and policy. Investment in surveillance strengthens laboratories and their ability to provide accurate diagnoses of bacterial infections to guide treatment decisions. It also links to the economics of food production, animal welfare, disease spillover risk, livelihoods, nutrition, and trade. The data surveillance provides has value outside its country of origin. It can be a global public good that may avert future AMR outbreaks.

The widespread value of vaccination is widely recognized [53] and should be proactively developed, including the consideration of new financial models based on cost-benefit analysis across sectors, and the how best to optimize public–private partnerships [54, 55].

Thus, surveillance can be effective at different levels of investment. Although an interconnected network of sites represents the optimal approach, even a single site is preferable to having no surveillance at all. However, maintaining high standards is essential. Private sector hospitals and laboratories also present important potential partners in expanding surveillance. In animal health, surveillance can also be integrated within disease control activities (eg, vaccination campaigns, abattoirs) or into routine wastewater monitoring programs. As a result, surveillance can be initiated with relatively modest resources [56].

The main costs of surveillance are laboratory hardware—a building with reliable utilities, equipment, consumables, such as specimen bottles and reagents, data management, salaries, training, and transport, including getting specimen samples to the national laboratory. Coupled with the hardware, is the “software” that include technical capacity to analyze, interpret, use, and communicate the relevant messages to difference audiences for varied short-, medium-, and long-term uses and the nuance to navigate the political, legal, social, and ethical dimensions that have been revealed as equally important to the AMR surveillance endeavor. The differences inherent in surveillance across the sectors need to be addressed by adequate, equitable multisectoral funding and the development of innovative surveillance strategies for the nonhuman sectors, embedding AMR surveillance into existing longstanding programs.

The Fleming Fund has invested considerably in laboratory infrastructure and equipment. The laboratories will only continue to provide good quality surveillance if consumables are constantly available, equipment is maintained, and staff expertise is preserved. Organizing at scale can reduce costs significantly through pooled procurement. The Fleming Fund has developed a basic costing tool to establish what funds are necessary to set up and run a hospital-based surveillance site, as well as assessing alternative means of gathering animal health data.

In-country financing for AMR surveillance comes mostly from government, with much smaller inputs from health insurance, the private health care sector, and private animal health laboratories and farms. Patients also often pay (out of pocket) for their own laboratory tests. The cost for passive sampling in animal health will most often be borne by the animal keeper. The role of government is crucial: a key goal is to get necessary AMR surveillance inputs included in annual budgets at both the national and subnational or local levels. In Kenya, a step forward for AMR surveillance in 2025–2026 was the fact that 8 counties (of 47), specified sums in AMR line items in their 2026 budgets. This shows the vital role of AMR public policy champions and is a sign of potential sustainability at least in those counties [57].

To ensure integration of future AMR programs, budgeted sustainability planning must happen from the outset, fully embedded in national institutions and aligned with existing financial and governance systems. The Fleming Fund demonstrated that although grants can build technical capability and develop high-quality surveillance tools, inevitably their long-term impact depends on domestic financing, institutional commitment, establishment of the necessary referral pathways, and staffing. Sustainability planning can include clear national responsibility for consumables and equipment maintenance (service contracts), integration of partner contributions into domestic budgets, adoption of surveillance strategies, standard operating procedures and data pathways, workforce development and retention plans, and embedding AMR governance structures within ministry mandates. As donor landscapes shift, sustainability must move from an aspiration to a core design principle underpinning all future AMR and One Health initiatives [33].

In the medium term, external funding will be necessary in many countries. The Global Fund offers the inclusion of AMR funding in its plans for the second half of the 2020s [28]. Indeed, the Global Fund calculates that spending on AMR has a good return on investment—at 1:28 [58]. Projections of major global economic losses from AMR in many sectors make AMR surveillance investment worthy of serious consideration.

## CONCLUSION

After identifying a key gap in global knowledge on AMR that could be filled with the establishment of surveillance systems, the Fleming Fund has drawn concerted effort and funding to this project over the past decade. The next decade now must focus on incorporating these capabilities and learnings into routine systems, recognizing that to address AMR requires a whole-system approach within the multiple diseases and sectors in which its impacts are felt. In the absence of increased, strategically targeted investment—whether through expanded domestic public financing, large-scale private sector engagement, or enhanced multilateral support—recent gains are unlikely to be sustained easily. Progress will require well-designed, targeted, and cost-effective investments to maintain current capacity while enabling further system strengthening.

Priority interventions may include the integration of private sector financing mechanisms within commercial animal production systems, the deployment of more rapid diagnostic technologies for clinical settings, and alignment with evolving health financing models, including private and publicly subsidized insurance schemes and other initiatives to improve access. Regardless of the approach, effective strategies to mitigate AMR over the next 25 years must be evidence-based, context-specific, economically efficient, and supported by clear communication to the public and the scientific, farming, and clinical communities. The Fleming Fund has helped to put AMR firmly on the agenda at global national and community levels, it has generated invaluable evidence and understanding on how to strengthen AMR surveillance systems. This has provided a legacy on which countries and regions can now build.
